# Public knowledge, belief, and preventive practices regarding dengue: Findings from a community-based survey in rural Bangladesh

**DOI:** 10.1371/journal.pntd.0011778

**Published:** 2023-12-07

**Authors:** Rajon Banik, Md. Saiful Islam, Mahfuza Mubarak, Mahmudur Rahman, Hailay Abrha Gesesew, Paul R. Ward, Md. Tajuddin Sikder

**Affiliations:** 1 Department of Public Health and Informatics, Jahangirnagar University, Savar, Dhaka-1342, Bangladesh; 2 College of Medicine and Public Health, Flinders University, Adelaide, South Australia, Australia; 3 Department of Epidemiology, School of Health Sciences, Mekelle University, Mekelle, Ethiopia; 4 Centre for Research on Health Policy, Torrens University Australia, Adelaide, South Australia, Australia; Al-Jouf University College of Pharmacy, SAUDI ARABIA

## Abstract

**Background:**

Dengue fever, the most prevalent mosquito-borne viral infection, is a recurrent public health threat in Bangladesh. Despite the government’s efforts, dengue outbreaks are on the upswing, and people’s knowledge, belief, and preventive practices regarding the disease at the rural community level are unclear.

**Objective:**

The objective of this study was to assess the level of knowledge, belief and preventive practices regarding dengue and associated factors among community people from rural Bangladesh.

**Methods:**

A cross-sectional survey was conducted involving 401 people using a convenient sampling technique from three unions of Savar from August to September 2021. Participants’ responses were collected through face-to-face interviews using a pre-tested structured questionnaire that included information related to socio-demographics, dengue-related knowledge, preventive practices, and the health belief model (HBM) constructs. Multiple linear regression analysis was performed to determine the factors associated with the knowledge and preventive practices of dengue.

**Results:**

Overall, participants (mean age = 33.47 ± 12.96 years; age range = 18–75 years) correctly answered 66.15% of the knowledge questions regarding dengue. Higher education, travel to dengue-risk regions, and self-efficacy under the HBM construct were all shown to be strongly associated with dengue knowledge. Regarding HBM constructs, about 80% of the participants perceived dengue as dangerous, but less than half (41.4%) believed themselves susceptible to dengue. Considering perceived barriers, 73.07% of the participants believed their residential area was not suitable for Aedes mosquito breeding. Nearly all (93.52%) believed they always kept their surrounding area clean as part of self-efficacy. Moreover, on average participants engaged in 53.69% of all dengue preventive practices. Being older, female, having a higher education, being a service holder, having a good quality of house structure, and perceived susceptibility as well as self-efficacy under the HBM construct were all factors in participants’ dengue prevention activities. Participants’ dengue preventative practices were shown to be significantly influenced by their knowledge.

**Conclusions:**

The findings of this study found a moderate level of knowledge regarding dengue among the participants. Regarding dengue prevention, although participants scored highly in several indicators, the overall preventive practices were not satisfactory. This suggests that there is a pressing need for expanded education outreach aimed at increasing public awareness of dengue and encourage preventive practices within rural communities in Bangladesh.

## Author summary

Dengue is a serious mosquito-borne disease of substantial public health concern, particularly in endemic countries like Bangladesh. It is vital to assess public knowledge and health beliefs regarding dengue fever in order to adopt efficient preventive practices and develop disease control strategies. The present study highlights dengue knowledge and preventive practices in relation to background characteristics and health beliefs among rural communities in Bangladesh. Findings from this study revealed that people had a moderate level of knowledge regarding dengue. Regarding health beliefs, people had high perceived severity, considering dengue as a dangerous and deadly disease, but their perceived susceptibility to dengue was low. Although people exhibited high self-efficacy, such as keeping their surrounding area clean, public involvement in dengue prevention practices was not satisfactory, except for some specific activities. Moreover, the dengue preventative practices of people were shown to be significantly influenced by their knowledge. Furthermore, evidence from this study emphasizes tailoring community-based health education campaigns to increase public awareness, which will improve health beliefs and encourage dengue prevention in Bangladesh.

## Introduction

Dengue cases are increasing dramatically worldwide, especially in countries located in the tropics and subtropics like Bangladesh [1,2]. Approximately 390 million dengue infections are estimated to occur annually, of which a quarter of the cases (67–136 million) will manifest clinically [1]. According to the World Health Organization, the number of dengue cases increased over 8-fold over the last two decades, from 505,430 cases in 2000 to over 2.4 million in 2010, and 5.2 million in 2019 [3]. Although the risk of infection exists in 129 countries [2], 70% of the actual encumbrance is in Asia [3].

**Fig 1:**
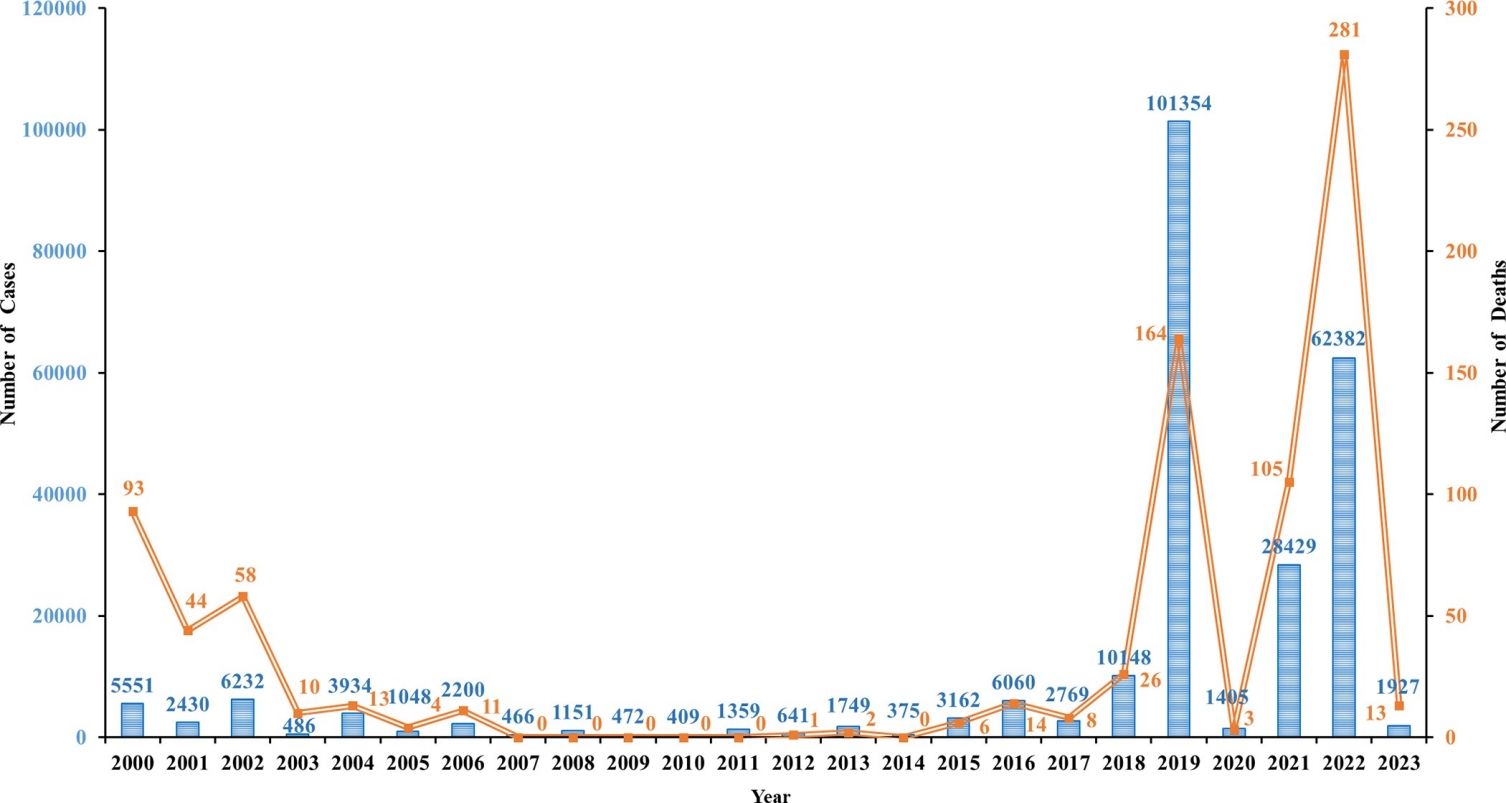
Trend of Dengue reported cases and deaths in Bangladesh 2000–2023 (May 30th).

Dengue is a mosquito-borne viral infection caused by any one of the 4 serotypes (DENV-1, 2, 3, and 4) of the dengue virus, which may appear as a fatal disease characterized by dengue hemorrhagic fever and dengue shock syndrome [4,5]. Dengue viruses are transmitted by female mosquitoes principally of the species *Aedes aegypti* and, to a lesser extent, *Aedes albopictus* [3]. Bangladesh, located in South Asia, has evolved into an appropriate spot for the dengue vector and its transmission [6]. The first case of dengue was discovered in Bangladesh in 1964 [7]. The sporadic cases and small outbreaks clinically suggest that the dengue occurred across the country from 1964 to 1999 but those were not officially reported [8,9]. In the year 2000, a severe outbreak of dengue occurred in Bangladesh with 93 mortality among 5551 morbidity cases [10]. Dengue occurrences dropped dramatically in succeeding years, reaching a low of 375 cases in 2014. However, in 2016, around 6100 dengue cases have been reported in Bangladesh [11]. Three years later, in 2019, Bangladesh experienced the highest annual dengue incidence ever reported with 101,354 cases and 164 deaths [12]. In 2020, Bangladesh reported 1405 dengue cases and only three confirmed dengue-related deaths [13]. However this number has increased substantially with 28,429 dengue cases and 105 dengue-related deaths in 2021, which was even higher in 2022 with a total of 62,382 dengue cases and 281 dengue-related death, and the 2022 outbreak is the second-largest outbreak since 2000 [14] (**Fig 1**). As of 30 May 2023, a total of 1927 dengue cases and 13 dengue-related deaths were reported in Bangladesh [15].

Dengue may be misclassified due to the wide range of disease signs and symptoms and a lack of adequate case definitions [16]. Therefore, it is highly probable that dengue cases may be substantially under-reported in Bangladesh given the weak surveillance of a struggling healthcare system [17]. Dengue fever is impacted by a number of variables, including uncontrolled population expansion, urbanization, and degradation in waste management systems [18], and lack of effective vector control [19]. Vector control is one of the most frequently applied methods of controlling or preventing dengue [20,21], which can be done by frequent fogging in endemic areas which is mostly done outdoors. However, the *Aedes aegypti* mosquito tends to rest hidden indoors, making it hard for insecticide to reach adult mosquitoes [22]. One of the few methods of dengue prevention is eliminating the breeding sites of dengue mosquitoes indoors and outdoors [23]. Human behavior is also an important contributor to creating breeding grounds for mosquitoes and sustaining mosquito populations [24,25]. The success of efforts in dengue prevention and control is mainly from improving public and household environmental sanitation, water supply, and alteration of human behavior towards dengue [26]. Furthermore, managing dengue outbreaks in tropical countries where temperatures remain favorable for mosquito breeding and viral replication around the year is a critical challenge [27]. Current vector-control in Bangladesh is a mandate of the local government engineering department. The city corporation or municipality is responsible for vector control activities including the elimination of breeding sites and larvicidal and adult mosquito control using different insecticides [28], for suppressing the mosquito vector activity at the optimal time in annual population fluctuations, in order to achieve the lowest biting population [27], when environmental conditions for emergence and transmission are most favorable, especially during the peak dengue period (August–September) [29]. The city corporations also carried out mass awareness campaigns–through television and other mass media and alerted building owners including buildings under construction to prevent water collection. Fines have been imposed on buildings where the Aedes larvae have been found [28]. Moreover, knowledge, beliefs, and practices about dengue is evident to have an impact on dengue prevention and control [17,25,30]. The HBM is by far the most commonly used theory which comprises several main constructs: perceived susceptibility, severity, benefits, barriers, self-efficacy to engage in a behavior and, cues to action [31]. In the context of dengue, the HBM provides a framework for understanding how to effectively structure messages and influence behavioral change [32]. A Malaysian study found perceived barriers to perform dengue prevention, perceived susceptibility to dengue fever as significant factors associated to dengue prevention practices [33]. Several demographic and behavioral factors significantly influence knowledge and attitude and practice regarding dengue. A recent study in Bangladesh showed the level of education as an independent predictor for both knowledge and awareness of dengue [34]. Another Bangladeshi study found a significant relationship between the education level and the occupation with the practice to prevent and control dengue [12]. Furthermore, successful participation largely depends on peoples’ knowledge, awareness, and attitude towards this disease [12,19]. A previous study found that Bangladeshi people are well aware of dengue [30]. Inadequate knowledge about dengue is a major risk factor faced in the elimination of dengue but lacks accurate knowledge about signs and symptoms, the transmission of dengue, and preventive practices can increase the spread of dengue fever among the Bangladeshi people [12,30]. However, evidence was found that higher knowledge did not necessarily result in the adoption of the recommended preventive behavior [12,34].

Therefore, further investigation is important to find effective preventive strategies as there is an ongoing challenge to ensure proper treatment and prevention options despite continued progress in dengue research throughout the world [35,36]. Although several research investigated public knowledge, attitude, and practice about dengue in Bangladesh [17,30,34] but there is a lack of evidence from Bangladeshi rural communities. Therefore, the study aimed at investigating the level of knowledge, health beliefs, and preventive practices regarding dengue and also identified associated factors in relation to background characteristics and HBM among the rural community people of Bangladesh.

## Methods and materials

### Ethics statement

This study maintained ethical standards to the highest possible extent, and informed consent was obtained from participants. This research was approved by the Biosafety, Biosecurity, and Ethical Review Board of the Jahangirnagar University, Savar, Dhaka-1342, Bangladesh [Ref No: BBEC, JU/M 2022/1(l)]. All responses were anonymous to ensure data confidentiality.

### Study area and population

**Fig 2:**
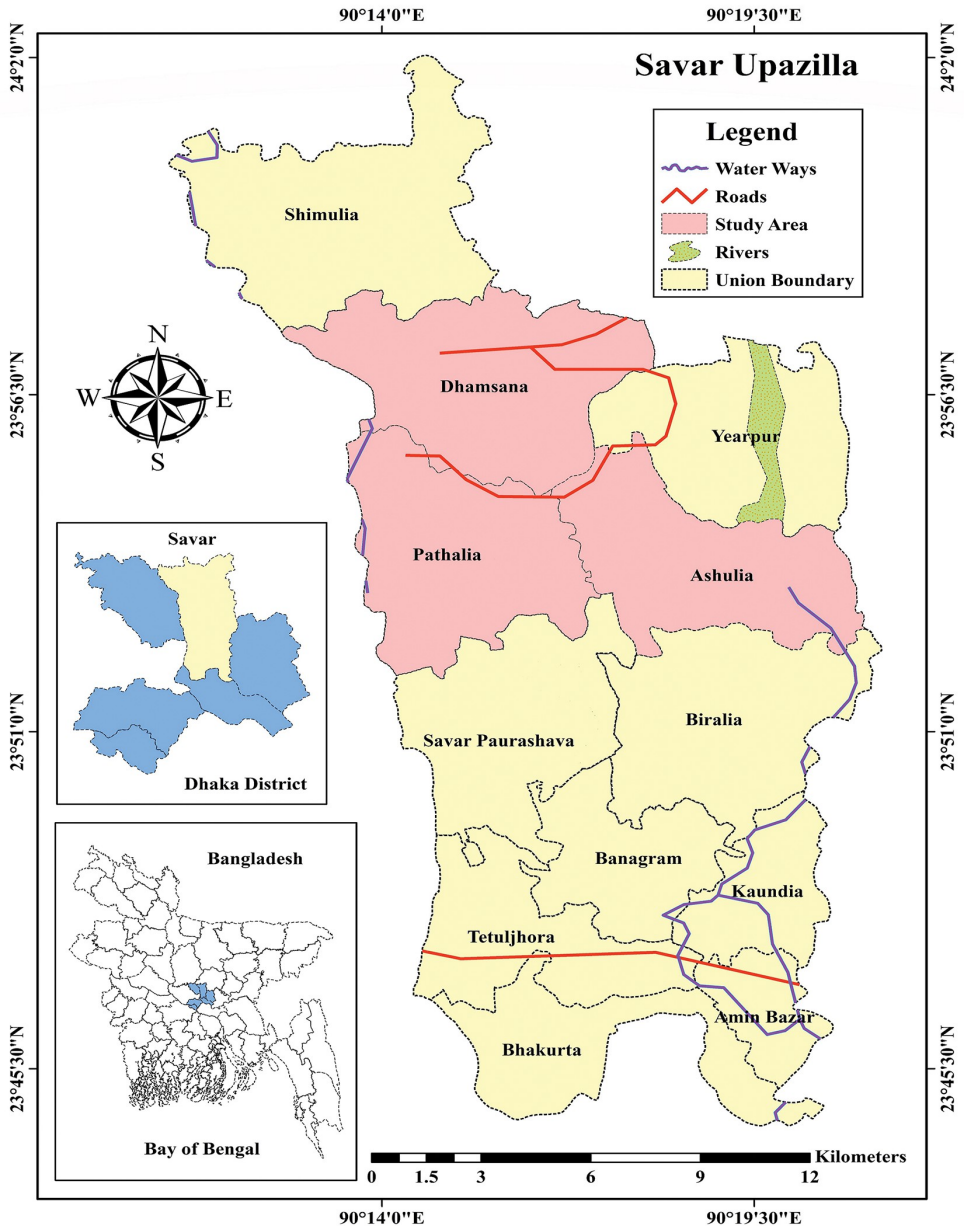
Spatial presentation of the areas included in the study (Source: The map was drawn from an open source data available at https://data.humdata.org/dataset/cod-ab-bgd).

A cross-sectional survey was carried out from August to September 2021, involving 401 Bangladeshi rural community people. The study area was Savar Upazilla, at a distance of about 24 kilometers to the northwest of Dhaka, the capital city of Bangladesh. Savar has experienced a rapid growth of population (7435 persons/km^2^) as well as expansion of urban areas and industrialization during the last twenty years [37]. In addition, there is no adequate drainage system (the exiting drain is also mostly clogged during heavy rains), and indiscriminate waste disposal [38], which creates natural and artificial water storage, serves as a main larval breeding habitat for *Aedes* mosquitoes (*Aedes aegypti* and *Aedes albopictus*) [39].The study employed a convenient sample technique. A total of 405 interviews were taken conveniently from three selected Unions [the smallest rural administrative and local government unit in Bangladesh [40]] of Savar Upazilla (Pathalia, Ashulia, and Dhamsana) (**Fig 2**). At the time of the survey, an eligible participant must be present in the household. Adults (over the age of 18) who had already lived in the area for at least one year prior to the research were eligible. Before the starting of the investigation the questionnaire was translated into Bangla and then translated back to English and pretested with 40 people to test the accuracy and validity. Data were collected by trained interviewers who visited door-to-door to invite people to participate in the survey. If a household had more than one eligible participant, one of them was chosen at random.

### Sampling

The sample size was calculated using the following formula:

$$n=\frac{z^{2}\times p\times (1-p)}{d^{2}}$$

$$n=\frac{1.96^{2}\times 0.5\times (1-0.5)}{0.05^{2}}=384.16\approx 384$$

Where, we considered *z* = 1.96 (95% confidence level), and *d* = 0.05 confidence interval as 0.05. The sample proportion was assumed as 0.5 since this value provide the maximum sample size. Hence, assuming 5% non-response rate the required sample size was 404. However, a total of 405 respondents completed the survey and after cleaning the incomplete responses 401 participants were taken for final analysis.

### Variables and measurement

Knowledge and preventive practices regarding dengue, described in detail below, are the response variables of the study. A self-reported structured questionnaire was developed after a review of previous studies [17,25,33,41,42]. The questionnaire consists of five main sections: i) socio-demographics, housing, surrounding environment, and dengue-related experience; ii) knowledge about dengue; iii) health belief model; and v) self-reported preventive practices.

Participants’ socio-demographic characteristics, including age, gender, marital status, education, occupation, family type, and monthly family income, were collected during the survey. Monthly family income was classified into four categories: less than 20,000 Bangladeshi Taka (BDT), 20,000–30,000 BDT, 30000–40000 BDT, and more than 40,000 BDT. Participants were also asked about housing type, density of vegetation/plants (not at all/low/moderate/much) and mosquitoes (not at all/low/moderate/severe) in the neighborhood and the presence or absence of a water reservoir. Questions were also asked to know the participants’ experience of dengue. Participants were asked about their history of dengue fever (yes/no), ever hospitalization due to dengue (yes/no), history of dengue infection of family members (yes/no), and traveling history in dengue risk areas (yes/no/don’t know).

The knowledge of dengue was assessed and consisted of 34 items divided into five subparts: 1) Knowledge about dengue and the Aedes mosquito, 2) Knowledge about the transmission of dengue, 3) Knowledge about the signs and symptoms of dengue, 4) Knowledge about preventive measures, and 5) Knowledge about treatment, curability, and precautionary measures for people infected with dengue. For each item, the response options were "yes," "no," or "don’t know." For the analyses, each correct response to a knowledge item has been assigned ‘1’ point, while ‘0’ points have been assigned to each incorrect or not-sure response that is deemed a correct response. Scores ranged from 0–34, where higher scores indicate greater knowledge about dengue fever [25,33].

Health beliefs regarding dengue fever were measured using the HBM constructs, which consists of four main parts: perceived threat, perceived barriers, self-efficacy, and a cue to action. Perceived Threat consists of two sub-parts which measure the participant’s susceptibility to contracting dengue fever and the severity of the dengue. Perceived barrier examines the perceptions of barriers to preventing dengue among participants. Self-efficacy is measured by the behavior of participants that successfully execute dengue prevention measures. Cues to action measure the mosquito problem, the frequency of fogging, community participation, and other things that affect an individual’s perception, which indirectly influences health-related behavior. All the responses were measured on a five-point Likert scale that ranged from 1 (strongly disagree) to 5 (strongly agree) [43]. The distribution of the HBM constructs is shown in **S1 Table**. However, for simplification, we presented the responses of the HBM as “disagree” (strongly disagree/disagree), "neutral," and “agree” (strongly agree/agree).

Self-reported preventive practices against dengue were sub-divided into three parts: prevention of mosquito breeding, prevention of mosquito bites, and prevention of dengue transmission. The questions were assessed using nine-item, seven-item, and one-item questions, respectively. The options for dengue prevention practices were "not at all", "rarely", "sometimes", "often", and "not applicable" and were assigned points of ‘0’, ‘1’, ‘2’, ‘3’, and ‘0’ respectively based on the number of applicable answers [33].

### Statistical analysis

All statistical analyses were performed using three software packages (Microsoft Excel 2019, SPSS version 25.0, and STATA version 15.0). Data cleaning, sorting, and coding were first performed using Microsoft Excel. Then, the excel file was imported onto the SPSS software for further analysis. Descriptive statistics (i.e., frequencies, percentages, means, and standard deviations) were computed using SPSS. Inferential statistics include conducting t-tests or one-way analyses of variance (ANOVA) to determine mean differences among variable groups and bivariate Pearson correlation was used for continuous variables. These analyses were done using SPSS. The variables that were significant (p < 0.05) in the bivariate analysis (t-test/ANOVA/Pearson correlations) with outcome variables (i.e., knowledge, attitudes, and practices) were then included in the multiple linear regression models to find out the associated factors of knowledge, attitudes, and practices, respectively using STATA. For all statistical tests, a p-value of less than 0.05 was considered statistically significant.

## Results

### Socio-demographic characteristics

A total of 430 individuals were approached, but only 405 were interviewed completely, giving a response rate of 94.19%. The profile of the respondents is given in Table 1, which summarizes the socio-demographic characteristics. A total of 401 participants comprised the study sample, and the majority (72.32%) were aged 18 to 39 years old. The mean (±SD) age of respondents was 33.47 (±12.96) years (age range: 18–75 years). Among the study participants, 62% were males, most of the participants were married (64%), and about 54% of the participants were undergraduate. Of the total sample, about 30% of the participants were students, followed by government and/or private service workers (termed here as ‘service holders’) (22%). Most of the participants belonged to nuclear families (78%) and had a monthly income of less than 30,000 BDT (69.82%) (Table 1).

**Table 1:** Background characteristics of participants.

Variables	Frequency, n (%)
***Demographic information***	
**Age**
	18–25 years	152 (37.91)
26–39 years	138 (34.41)
≥40 years	111 (27.68)
**Gender**
	Male	250 (62.34)
Female	151 (37.66)
**Marital status**
	Unmarried	143 (35.66)
Married	258 (64.34)
**Education**
	No formal education	39 (9.73)
Primary	31 (7.73)
Secondary	105 (26.18)
Intermediate	45 (11.22)
Graduate	147 (36.66)
Post-graduate	34 (8.48)
**Occupation**
	Housewife	75 (18.7)
Student	120 (29.93)
Service holder	93 (23.19)
Businessman	71 (17.71)
Day labor	26 (6.48)
Others	16 (3.99)
**Family type**
	Nuclear	313 (78.05)
Joint	88 (21.95)
**Monthly family income**
	<20,000 BDT	143 (35.66)
20,000–30,000 BDT	137 (34.16)
30,000–40,000 BDT	73 (18.2)
>40,000 BDT	48 (11.97)
***Housing*, *surrounding environment and experience of dengue***	
**Type of house**
	Building	158 (39.4)
Semi-building	81 (20.2)
Tin shed	154 (38.4)
Others	8 (2)
**Density of vegetation/plants in the neighborhood**
	Not at all	15 (3.74)
Low	112 (27.93)
Moderate	201 (50.12)
Much	73 (18.2)
**Water reservoirs around the house**
	Yes	205 (51.12)
No	196 (48.88)
**Quantity of mosquito in neighborhood**
	Not at all	6 (1.5)
Low	66 (16.46)
Moderate	215 (53.62)
Severe	114 (28.43)
**Dengue history**
	Yes	11 (2.74)
No	390 (97.26)
Hospitalization due to dengue*
	Yes	4 (36.36)
No	7 (63.64)
**Dengue history of family members**
	Yes	24 (5.99)
No	377 (94.01)
**Traveling in dengue risk areas**
	Yes	83 (20.7)
No	232 (57.86)
Don’t know	86 (21.45)

*Sample was limited to participants who had experience of dengue (n = 11)

### Housing, surrounding environment, and dengue-related experience

Of the total responses, building was reported as the most common housing type (39.4%). The moderate density of vegetation/plants surrounding their houses was reported by the majority of the participants (50.12%). More than half the participants (51.12%) reported having water reservoirs around their houses, and mosquito density in the neighborhood was reported as severe by 28.43% of the participants. Only a few (2.74%; n = 11) participants had previously been infected with dengue, and among them, 36.36% (n = 4) of participants had been hospitalized. Likewise, a minority of participants (5.99%) reported having a family member previously infected with dengue and 57.86% reported not traveling in a dengue risk area (Table 1).

### Knowledge

The mean knowledge score for participants was 22.49 (SD: 6.27; range 0–34). On average, participants correctly answered 66.15% of the knowledge questions. Table 2 shows that 66.33% of the participants correctly answered that dengue is a viral disease, and a higher proportion of the participants (93.27%) correctly answered that dengue fever is transmitted by a mosquito. Most participants (78.3%) knew that dengue fever is mainly transmitted by the Aedes mosquito in Bangladesh. Only a minority of the participants (11.12%) knew that the Aedes mosquitoes do not live in places with a lot of plants. The majority of the respondents (75.06%) knew that Aedes mosquitoes usually bite during dawn and dusk. A modest number of participants (70.57%) correctly answered that dengue can be spread by an Aedes mosquito biting an infected person and then biting a healthy person. Fever was the most consistent response (91.02%) when asked about the common symptoms of dengue. Beside fever, 60–75% of participants knew signs and symptoms of dengue, e.g., rash, nausea/vomiting, joints pain, headache. Pain in the eyes (49.38%) and bleeding (36.41%) were fewer known signs and symptoms by the participants. More than half of the participants (61.1%) had the misconception that one can get dengue only once. Dengue fever can be avoided by using preventive measures. When asked about dengue prevention, the most frequent responses were the weekly change of stagnant water (pet bowls, vases) (95.76%) and the removal of mosquito breeding sites (94.76%). When respondents were asked about the treatment, very few (19.45%) correctly answered that there is no specific treatment for dengue, but the majority of the participants knew about practices such as taking rest (80.55%) and drinking adequate water (78.05%) as supportive practices for people infected with dengue.

**Table 2:** Overall knowledge of participants about dengue (correct responses).

Knowledge items	Frequency, n (%)
***Knowledge about dengue and Aedes mosquito***
	Dengue is a viral disease (True)	266 (66.33)
Dengue is spread through mosquito bites (True)	374 (93.27)
Dengue is transmitted by Aedes mosquito (True)	314 (78.3)
Aedes mosquitoes have black and white stripes on their body (True)	247 (61.6)
Aedes mosquitoes breed in dirty and unclean water (True)	285 (71.07)
Aedes mosquitoes prefer to stay indoors or in buildings as compared to natural wetlands (True)	230 (57.36)
Aedes mosquitoes prefer to live in places where there are many plants (False)	45 (11.22)
Aedes mosquitoes usually bite during the sunrise or sunset (True)	301 (75.06)
***Knowledge about the transmission of dengue***
	Biting an infected person and then biting a healthy person (True)	283 (70.57)
Infected person to a healthy person through direct contact (False)	267 (66.58)
Through air (False)	279 (69.58)
Through blood transfusion (True)	258 (64.34)
In case of any kind of injury through infected needle (True)	245 (61.1)
By using any the food/clothes of the infected person (False)	226 (56.36)
One can get dengue fever once (False)	156 (38.9)
Dengue epidemic mainly occurs in rainy season (True)	300 (74.81)
***Sign and symptoms of dengue***
	High fever (True)	365 (91.02)
Rash (True)	283 (70.57)
Pain in muscle (True)	287 (71.57)
Nausea/vomiting (True)	287 (71.57)
Joint pains (True)	243 (60.6)
Headache (True)	302 (75.31)
Bleeding in the gums and nose (True)	146 (36.41)
Pain in the eyes (True)	198 (49.38)
***Knowledge about prevention***
	Weekly removal of mosquito breeding sites (True)	380 (94.76)
Weekly change of stagnant water (pet bowls, vases) (True)	384 (95.76)
Put abate/chemical in water containers (True)	212 (52.87)
Covering water containers (True)	356 (88.78)
Emptying or drying out containers around the house (True)	367 (91.52)
Proper disposal of items that can retain water (True)	365 (91.02)
***Knowledge about treatment*, *curability and precautionary measures for people infected with dengue***	
No specific medication for dengue (True)	78 (19.45)
Take rest (True)	323 (80.55)
Drink adequate water (True)	313 (78.05)
Vaccination (True)	97 (24.19)

Knowledge was significantly associated with age, marital status, education, occupation, family type, monthly income, house type, and traveling in dengue risk areas, as well as positively correlated with self-efficacy (Tables 4 & 5*)*. Of these, having a higher education, travelling in dengue risk areas, and self-efficacy were significantly associated with knowledge regarding dengue in the multiple regression analysis (Table 6).

### Health beliefs

**Fig 3:**
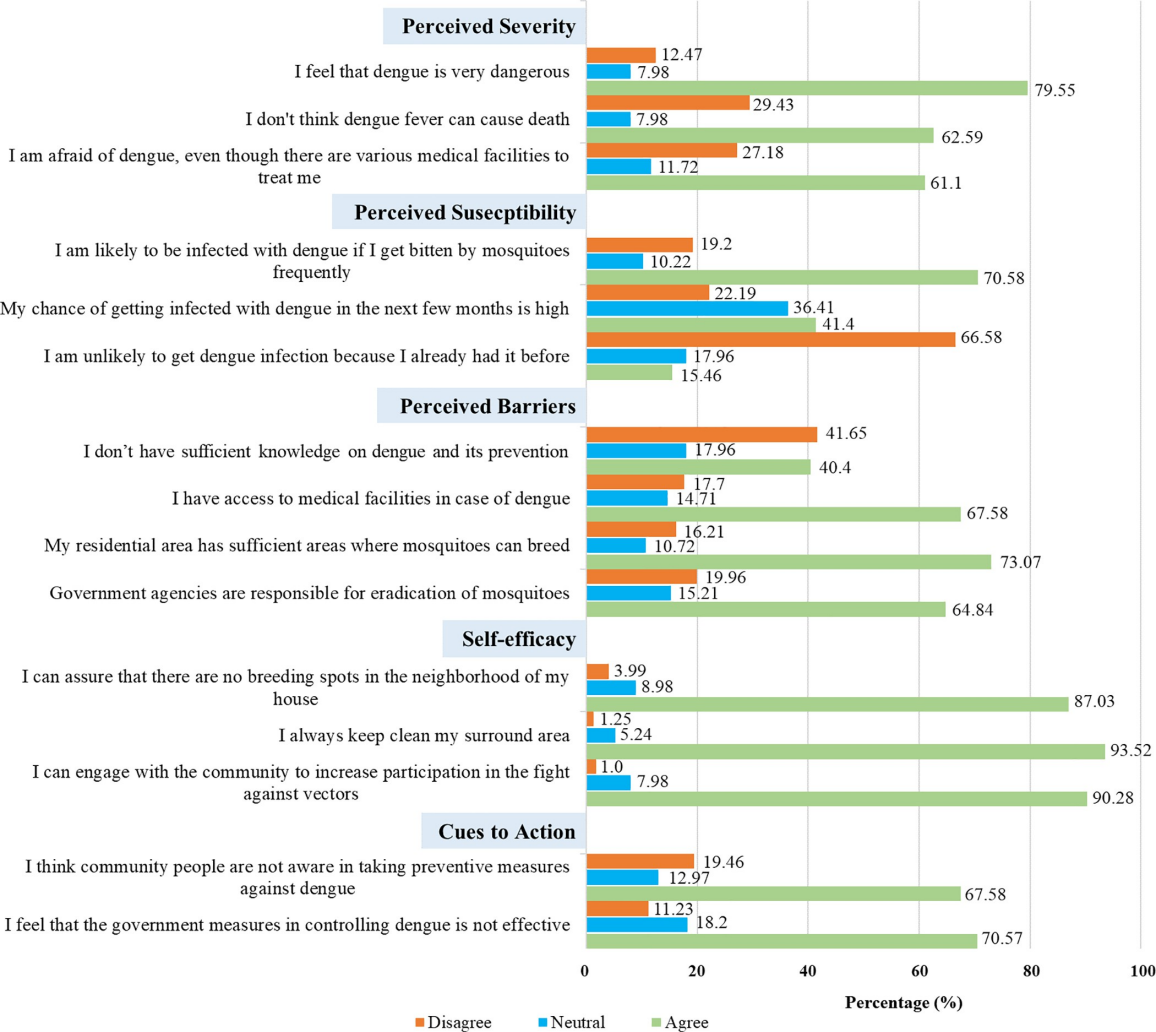
Participants belief regarding dengue based on HBM.

The distribution of each item of the HBM is presented in Fig 3. The mean (SD) rating of the perceived severity of dengue was 10.53 (SD: 2.13; range 3–15). Items regarding the perceived severity under the HBM construct, 79.55% of the participants perceived that dengue is very dangerous, but interestingly, only 29.43% believed that dengue fever can cause death. Furthermore, 61.1% of respondents agreed that they are still concerned about getting dengue even though various medical facilities are available in their surroundings. On the perceived susceptibility under the HBM construct, the mean score was 9.05 (SD: 2.13; range 3–15). Among the participants, 70.58% were worried about being infected with dengue if they got bitten by mosquitoes. However, less than half (41.4%) believed they could get infected with dengue in the next few months, and 15.46% of them considered them less susceptible to dengue because they had previously been infected with it. On the other hand, believing that their residential area is not suitable for Aedes mosquito breeding (73.07%), accessibility of medical services (67.58%), and only government agencies are responsible for mosquitoes eradication (64.84%) were the main perceived barriers [mean: 13.66 (SD: 2.14; range 4–20)] to dengue prevention among the participants. Total mean score of self-efficacy was 12.09 (SD: 1.62; range 3–15) and regarding items almost all (93.52%) the participants were agreed that they always keep their surrounding area clean and 90.28% believed they could engage with the community to increase participation and mobilization in the fight against vectors. With regards to cues to action, 67.58% perceived community people are not aware in taking preventive measures against dengue and only 11.23% perceived that the government measures in controlling dengue is effective and the mean score of cues to action was 7.32 (SD: 1.57; range 2–10).

### Preventive practices

Table 3 shows that most of the participants practiced covering all water used for storing in or outside the house (98.50%) and changing stored water in flower vases, drip trays, or pails (93.77%). Most of them also practiced proper disposal of household garbage (96.76%). It was also noted that 90.52% of the participants practice cleaning the areas surrounding their houses frequently as one of the reasons to prevent dengue. Only a minority of the participants (29.68%) use Abate or chemicals in water storage containers to prevent dengue mosquito breeding, and 20.70% of the participants use mosquito repellent on their bodies to prevent mosquito bites. On average, participants engaged in 53.69% of all dengue preventive practices, with a mean practice score of 24.16 (SD: 6.53; range 0–45).

**Table 3:** Practices of the participants for prevention of dengue.

Practice items	Frequency, n (%)
***Prevention of mosquito breeding***	
Cover all water used for storing in or outside the house	395 (98.50)
Change stored water in flower vases, drip tray or pails	376 (93.77)
Put abate or chemical in water storage containers	119 (29.68)
Examine for mosquito eggs in containers for storing water	222 (55.36)
Clear out debris that may block water flow in drain or roof gutters	318 (79.30)
Proper disposal of items that can collect rain water	338 (84.29)
***Prevention of mosquito bite***	
Sleep in mosquito net of have mosquito screens on windows	382 (95.26)
Use mosquito coil, electrical mosquito mat, liquid vaporizer	353 (88.03)
Spraying dark places with an insecticidal spray	132 (32.92)
Use mosquito repellent on body	83 (20.70)
Wear long-sleeved shirts and pants to avoid mosquito bites	115 (28.68)
Wear bright color clothes to avoid mosquito bites	53 (13.22)
***Prevention of dengue transmission***	
Take measure to prevent mosquito from biting a dengue patient	230 (57.36)
***Cues to action***	
Proper disposal of household garbage	388 (96.76)
Clean up surrounding house area	363 (90.52)

Participants preventive practice was significantly associated with age, gender, education, occupation, house type, water reservoirs around the house, density of mosquitoes in the neighborhood, and traveling in dengue risk areas, as well as positively correlated with perceived severity, perceived susceptibility, self-efficacy, and knowledge of dengue (Table 4 and 5). Of these, being older, being female, having higher education, being a service holder, having a good quality of house structure, perceived susceptibility, and self-efficacy were significantly associated with preventive practices regarding dengue in the multiple regression analysis (Table 6).

**Table 4:** Association of participants’ knowledge and practices regarding dengue with the background characteristics.

Variables	*Knowledge*			*Practices*		
	Mean (SD)	t/F	*p*-value	Mean (SD)	t/F	*p*-value
**Age**						
18–25 years	24.09 (4.95)	13.88	**<0.001**	22.97 (6.74)	4.19	**0.016**
26–39 years	22.64 (5.85)			25.01 (6.45)		
≥40 years	20.1 (7.56)			24.72 (6.14)		
**Gender**						
Male	22.94 (6.05)	3.50	0.062	23.23 (6.64)	13.88	**<0.001**
Female	21.74 (6.57)			25.7 (6.06)		
**Marital status**						
Unmarried	24.37 (4.69)	21.10	**<0.001**	23.73 (7)	0.96	0.327
Married	21.44 (6.78)			24.4 (6.25)		
**Education**						
No formal education	13.85 (7.31)	48.00	**<0.001**	24 (5.97)	2.49	**0.031**
Primary	18.35 (6.3)			21.19 (6.29)		
Secondary	20.56 (5.57)			23.98 (5.93)		
Intermediate	24 (3.88)			22.87 (7.43)		
Graduate	25.62 (3.94)			25.16 (6.6)		
Post-graduate	26.56 (3.47)			24.97 (6.86)		
**Occupation**						
Housewife	19.55 (6.79)	23.51	**<0.001**	24.75 (5.43)	2.77	**0.018**
Student	25.33 (3.89)			23.6 (6.62)		
Service holder	24.47 (4.5)			24.85 (6.76)		
Businessman	19.73 (6.87)			25.11 (6.78)		
Day labor	16.46 (7.77)			20.23 (5.87)		
Others	25.44 (3.83)			23.69 (7.25)		
**Family type**						
Nuclear	23.08 (5.81)	13.33	**<0.001**	24.03 (6.36)	0.58	0.447
Joint	20.36 (7.33)			24.63 (7.11)		
**Monthly income**						
<20,000 BDT	20.83 (6.78)	7.11	**<0.001**	23.55 (6.49)	0.87	0.455
20,000–30,000 BDT	22.61 (5.88)			24.72 (6.54)		
30,000–40,000 BDT	24.32 (4.66)			23.95 (6.58)		
>40,000 BDT	24.31 (6.7)			24.69 (6.59)		
**House type**						
Building	24.03 (5.67)	9.53	**<0.001**	24.46 (6.51)	4.00	**0.008**
Semi-building	23.31 (4.95)			24.9 (5.86)		
Tin shed	20.47 (7.04)			23.83 (6.41)		
Others	22.38 (3.54)			16.88 (11.1)		
**Density of vegetation/plants**						
Not at all	22.73 (5.61)	1.61	0.187	24.87 (5.54)	0.32	0.814
Low	21.59 (6.32)			23.74 (7)		
Moderate	23.13 (5.87)			24.18 (6.46)		
Much	22.04 (7.21)			24.59 (6.24)		
**Water reservoirs around the house**						
Yes	22.95 (6.26)	2.27	0.133	23.41 (6.62)	5.48	**0.020**
No	22.01 (6.26)			24.93 (6.36)		
**Density of mosquito in neighborhood**						
Not at all	21.17 (9.83)	1.26	0.290	21.83 (6.37)	3.63	**0.013**
Low	22.65 (5.23)			25.03 (6.31)		
Moderate	22.94 (6.61)			23.26 (6.3)		
Severe	21.6 (5.91)			25.47 (6.86)		
**Dengue history**						
Yes	23.45 (4.5)	0.27	0.604	25.55 (10.04)	0.51	0.475
No	22.46 (6.31)			24.12 (6.42)		
**Hospitalization due to dengue**						
Yes	22.12 (4.26)	0.87	0.373	27.25 (9.48)	1.04	0.332
No	24.75 (5.32)			21.25 (9.91)		
**Dengue history of family members**						
Yes	24.08 (5.47)	1.66	0.198	23.21 (7.82)	0.54	0.464
No	22.38 (6.31)			24.22 (6.45)		
**Traveling in dengue risk areas**						
Yes	25.35 (5.36)	15.35	**<0.001**	23.87 (6.81)	9.19	**<0.001**
No	21.18 (6.52)			25.16 (6.19)		
Don’t know	23.24 (5.34)			21.72 (6.56)		

**Table 5:** Association among participants’ knowledge, practices, and health belief model constructs.

Variables	Skewness (SE)	Kurtosis (SE)	Mean (SD)	Range	1	2	3	4	5	6	7
1. Perceived severity	-0.48 (0.12)	0.27 (0.24)	10.53 (2.13)	3–15	⸻						
2. Perceived susceptibility	-0.47 (0.12)	-0.05 (0.24)	9.05 (2.13)	3–15	0.36***	⸻					
3. Perceived barriers	-0.53 (0.12)	0.84 (0.24)	13.66 (2.14)	4–20	0.14**	0.14*	⸻				
4. Self-efficacy	-1.09 (0.12)	6.2 (0.24)	12.09 (1.62)	3–15	-0.08	<0.01	0.01	⸻			
5. Cues to action	-0.46 (0.12)	0.09 (0.24)	7.32 (1.57)	2–10	0.03	0.04	0.17**	0.19***	⸻		
6. Knowledge	-1.1 (0.12)	1.19 (0.24)	22.49 (6.27)	0–34	-0.08	0.03	-0.06	0.15**	0.05	⸻	
7. Practice	-0.3 (0.12)	-0.13 (0.24)	24.16 (6.53)	0–45	0.17**	0.23***	-0.02	0.14**	-0.07	0.21***	__

Note

SE = standard error; SD = standard deviation

*p<0.05

**p<0.01

***p<0.001

**Table 6:** Results of multiple regression analysis predicting knowledge and practices regarding dengue among the participants.

Variables	*Knowledge*					*Practices*				
	B	SE	t	β	*p*-value	B	SE	t	β	*p*-value
**Age**										
18–25 years	Ref.					Ref.				
26–39 years	-0.18	0.87	-0.21	-0.01	0.837	1.90	1.00	1.91	0.14	0.057
≥40 years	0.40	0.97	0.41	0.03	0.683	2.93	1.08	2.72	0.20	**0.007**
**Gender**										
Male						Ref.				
Female	⸻	⸻	⸻	⸻	⸻	2.90	0.82	3.53	0.22	**<0.001**
**Marital status**										
Unmarried	Ref.									
Married	1.53	0.95	1.61	0.12	0.109	⸻	⸻	⸻	⸻	⸻
**Education**										
No formal education	Ref.					Ref.				
Primary	3.81	1.23	3.11	0.16	**0.002**	-2.61	1.47	-1.77	-0.11	0.077
Secondary	6.20	1.01	6.13	0.44	**<0.001**	1.23	1.21	1.02	0.08	0.310
Intermediate	8.81	1.26	7.02	0.44	**<0.001**	0.71	1.48	0.48	0.03	0.631
Graduate	10.25	1.20	8.57	0.79	**<0.001**	3.64	1.42	2.57	0.27	**0.011**
Post-graduate	11.63	1.39	8.39	0.52	**<0.001**	2.18	1.64	1.33	0.09	0.185
**Occupation**										
Housewife	Ref.					Ref.				
Student	1.54	1.27	1.21	0.11	0.225	1.04	1.43	0.73	0.07	0.469
Service holder	0.64	0.88	0.73	0.04	0.468	2.71	1.23	2.20	0.16	**0.029**
Businessman	-1.55	0.86	-1.79	-0.09	0.074	0.49	1.22	0.40	0.03	0.691
Day labor	-1.30	1.16	-1.13	-0.05	0.260	-0.64	1.51	-0.42	-0.02	0.672
Others	2.59	1.44	1.79	0.08	0.074	-0.14	1.85	-0.07	<-.01	0.941
**Family type**										
Nuclear	Ref.									
Joint	-1.10	0.61	-1.80	-0.07	0.073	⸻	⸻	⸻	⸻	⸻
**Monthly income**										
<20,000 BDT	Ref.									
20,000–30,000 BDT	0.42	0.93	0.45	0.03	0.656	⸻	⸻	⸻	⸻	⸻
30,000–40,000 BDT	0.14	0.88	0.16	0.01	0.877	⸻	⸻	⸻	⸻	⸻
>40,000 BDT	0.19	0.94	0.20	0.01	0.838	⸻	⸻	⸻	⸻	⸻
**House type**										
Building	Ref.					Ref.				
Semi-building	0.32	0.71	0.45	0.02	0.655	0.85	0.82	1.03	0.05	0.303
Tin shed	-0.64	0.64	-1.01	-0.05	0.315	0.13	0.75	0.18	0.01	0.859
Others	-0.53	1.85	-0.29	-0.01	0.774	-4.67	2.23	-2.10	-0.10	**0.037**
**Water reservoirs around the house**										
Yes						Ref.				
No		⸻	⸻	⸻	⸻	1.07	0.64	1.67	0.08	0.095
**Density of mosquito in neighborhood**										
Not at all						Ref.				
Low	⸻	⸻	⸻	⸻	⸻	2.63	2.55	1.03	0.15	0.302
Moderate	⸻	⸻	⸻	⸻	⸻	1.26	2.48	0.51	0.10	0.612
Severe	⸻	⸻	⸻	⸻	⸻	3.42	2.53	1.35	0.24	0.178
**Traveling in dengue risk areas**										
Yes	Ref.					Ref.				
No	-1.50	0.66	-2.28	-0.12	**0.023**	1.00	0.80	1.25	0.08	0.213
Don’t know	0.54	0.78	0.70	0.04	0.483	-0.65	0.94	-0.69	-0.04	0.492
**Health Belief Model**										
Perceived severity	⸻	⸻	⸻	⸻	⸻	0.13	0.16	0.84	0.04	0.403
Perceived susceptibility	⸻	⸻	⸻	⸻	⸻	0.53	0.15	3.46	0.17	**0.001**
Self-efficacy	0.46	0.15	2.97	0.12	**0.003**	0.65	0.19	3.52	0.16	**<0.001**

## Discussion

As Bangladesh is located in Southeast Asia, vector-borne diseases such as dengue fever are considered a severe health threat [44]. Overcrowding and uncontrolled urbanization have been identified as critical factors in the spread of mosquito-borne diseases such as dengue fever [7]. In the occurrence of dengue epidemics and the implementation of control measures, socio-demographic variables, as well as community awareness and practice, are crucial [45]. This study was conducted to assess the rural community’s knowledge, beliefs, and preventive practices regarding dengue. The present study found that more than three-fifths (66.15%) of the study population were knowledgeable about dengue, which is comparable to a recent study in Bangladesh [34]. However, a hospital-based survey in Bangladesh’s Dhaka city observed that just 52% of individuals were knowledgeable about dengue fever [17]. A substantial majority of the participants were aware that dengue is transmitted by mosquito bites, which is consistent with earlier studies conducted in Bangladesh [34], and Malaysia [33]. Although the participants in this survey had a fair understanding of dengue, there are still some misconceptions concerning the disease’s vector, breeding grounds, and route of transmission. More than one-fifth of the participants didn’t know that the Aedes mosquito is the carrier of dengue fever. Approximately thirty percent of the participants reported that the most common breeding sites for dengue mosquitoes are dirty and unclean water, such as sewer drains, and they prefer to live in places where there are many plants. Our findings on dengue knowledge are comparable to similar knowledge, attitude and practice (KAP) research findings in India [46], Malaysia [47], Indonesia [48], and Yemen [49]. More crucially, 75% of respondents reported Aedes mosquito bites during sunrise and sunset. This finding was consistent with findings from earlier research conducted in other countries [23,50]. When compared to the findings of previous research in Dhaka [30,51], findings from present study indicates that community members have a better understanding of dengue fever. This higher trend in knowledge might be attributed to health officials’ increased educational campaigns in response to ongoing outbreaks and an exponential growth in the number of dengue cases in recent years [12,52,53]. According to many of the interviewees, the most prevalent breeding grounds for dengue mosquitoes are dirty and unclean water, such as sewage drains. Our findings on DF knowledge are comparable to similar KAP research findings previously done in India [46], Malaysia [47], Indonesia [48], and Yemen [49].

More than ninety percent of the participants in this survey identified fever as one of the common and obvious signs and symptoms of the illness, which is consistent with previous research [17,33,54]. Although, most of the participants were correctly identified, still some were less knowledgeable about symptoms like joint pains, headaches, bleeding in the gums and nose, which are the main signs and symptoms of dengue. Furthermore, the majority of the participants in this research had never had dengue fever or had a family member infected with the disease, which is why they did not identify the other typical symptoms of dengue. Another challenge is that dengue fever is readily mistaken for other frequent causes of fever, including influenza and typhoid [54], or even COVID-19 [55]. As a result, there is a need for education to help people distinguish between dengue and other diseases so that they can seek prompt treatment and avoid unwarranted death as a result of dengue. In the present study, more than half of the study participants thought that dengue can be transmitted from an infected person to a healthy person through direct contact, which is comparable to a previous study performed among Nepalese people [56]. Such misunderstandings may lead to the notion that dengue is an unavoidable disease for people, because mosquito avoidance alone is not enough to prevent the disease, as per community people.

According to the current study, more than a third of the participants (64.39%) believe the dengue virus may be contracted through blood transfusion. However, in actual, viral infection following a blood or organ transplant is rare [57]. In Singapore, for example, the risk of receiving dengue-infected blood transfusions was estimated to be 1.625–6/10,000 in 2005 [58]. Surprisingly, only a few of the participants knew that there was no specific medication for dengue, which corresponds to previous findings [33]. However, about 80% of the people agreed that they had known about the primary treatments like getting plenty of bed rest and drinking lots of water. A similar finding was reported in a study from Jamaica [54].

Multivariate analysis found education level was significantly associated with participants’ knowledge regarding dengue, which was also found in earlier studies [59,60], reporting the higher the education level, the better the knowledge of dengue.

Monthly family income was found to be significantly associated with knowledge about dengue. Some studies on dengue have also demonstrated a direct relationship between income class and knowledge [60,61]. People with a higher economic status may have more access to and appreciation for trustworthy information. These findings suggest that more effective population awareness campaigns, aimed at those in lower income categories, should be conducted. There was also a link between age, marital status, and dengue knowledge scores. Other studies have also described significant associations between good knowledge scores of dengue with being married [5,47], increasing age [47]. Traveling to dengue-risk areas was also revealed to be a predictor of dengue knowledge. This is not unexpected given that visitors are not only at danger of getting dengue fever, but they also contribute to the disease’s transmission to non-endemic areas [47]. As a result, they may have a better understanding of dengue. Self-efficacy, as evaluated by the HBM construct, was also a significant influence on the research participants’ understanding of dengue fever. Similar findings were discovered in a Malaysian study [33]. Low self-efficacy and perceived benefit of continued dengue prevention practices may result in lack of concerted action against dengue prevention in their neighborhood.

Despite a moderate degree of knowledge about dengue, there was still a lack of conscious desire regarding some important aspects of dengue prevention among the participants. Based on the mean practice score, on average, participants engaged in just half of the dengue preventive practices. The majority of the participants covered all the water used for storage inside or outside the house and changed stored water in flower vases, drip trays, or pails, which is consistent with research performed among Malaysian community people [33]. A little more than half of the participants, on the other hand, looked for mosquito eggs in water storage containers. Indoor containers were discovered to contain immature Aedes mosquito eggs, indicating that Aedes mosquitos had adapted to reproducing indoors due to easy access to blood [33]. As a result, not only should efforts be made to destroy mosquito eggs to prevent dengue fever from spreading outside, but also within the home. Only a small percentage of the participants were aware of the use of Abate, a mosquito repellent that may be used in water containers to inhibit mosquito reproduction. In Thailand, using Abate to decrease Aedes aegypti in water holding containers was proven to be effective [62]. However, less than a quarter of the participants inappropriately disposed of unused water-holding containers. These containers are potential breeding sites for mosquitoes that may transmit infections, including dengue [16].

Females were found to be a predictor of excellent preventative measures in the current investigation. This might be due to the fact that women, especially housewives, spend a lot of time at home and are more inclined to clean the house [63]. Participants with higher education levels, particularly graduates, were significantly associated with preventive practices against dengue which is consistent with a previous study [64]. This suggests that educational attainment may be linked to the population’s ability to integrate and coordinate efforts to restrict transmission. Our study’s multivariate findings provide a foundation for developing educational and health messaging for health behavioral change interventions based on HBM components. Higher perceived vulnerability to dengue was linked to more stringent dengue preventative behaviors, which was consistent with prior findings [33,59]. This might be due to the fact that the majority of the participants are ignorant of the hazards of dengue fever and have never had it. In addition, self-efficacy for dengue prevention was shown to be a predictor of dengue preventative practices. In predicting the intention to carry out the dengue preemptive measures, self-efficacy showed the greatest predictive power compared to the other predictor factors [41]. An association has been found between knowledge and preventive practices. These findings are similar to those reported from Bangladesh [34] and Nepal [56]. Therefore, education efforts must emphasize the equal risk of contracting the disease in order to raise awareness among those who are ignorant of the significant threat posed by dengue fever. Testimonials and campaigns from families who have lost a loved one to dengue disease can be utilized.

Inadequate preventive practices, a high human population density, and a favorable habitat for Aedes reproduction in Bangladesh, particularly in big cities, may all contribute to a higher risk of transmission. Other research has indicated high levels of knowledge but low levels of practice, and these findings on practice levels are comparable. [62,65]. In fact, if the gap between knowledge and practice isn’t decreased, then it will be an important challenge for controlling dengue and Aedes populations.

### Strengths and limitations

The strength of this study is that it intends to assess public knowledge and preventative behaviors about dengue in rural communities of Bangladesh, where the disease is becoming more prevalent. The findings will serve as guides to health care planners for better strategic planning of dengue control. Furthermore, the study includes a rigorous sampling approach and a diverse variety of respondents to represent the comprehensive perspective of a rural community. Another strength of this study is its face-to-face interview approach. The present study has certain limitations. First, the cross-sectional design precludes the identification of causal relationships between variables. Second, the research may only capture a snapshot of information of the participants and hence cannot be generalized to other groups; the results may vary over time. Third, convenience sampling was employed to select the study area and respondents in this study, which may have resulted in some bias in our respondents’ representation. Finally, even though the survey was anonymous, the responses to the questionnaire may not reflect actual beliefs and practices since respondents may seek to provide socially acceptable responses.

## Conclusions

The results suggest that participants had a moderate level of knowledge regarding dengue and Aedes mosquitoes, modes of transmission, signs and symptoms, as well as preventive strategies. Considering health beliefs, although respondents had higher perceived severity and perceived barriers to dengue prevention, their perceived susceptibility to dengue was low. Interestingly, respondents believed they had high self-efficacy to fight dengue. On the other hand, participants engaged in approximately half of all preventive practices towards dengue. Majority of the participants exhibited a relatively high level of consciousness for several aspects of dengue preventive practices, such as the elimination of mosquito breeding sites, precautions to avoid mosquito bites, and cues to action, but the overall score of dengue prevention practices was not satisfactory. Practical, family-oriented, and community-based health education campaigns must be tailored to increase dengue knowledge, deter negative community beliefs, and encourage dengue preventative practices in rural communities of Bangladesh.

## Supporting information

S1 Table Distribution of the HBM constructs. (DOCX)

S1 File Full Questionnaire. (DOCX)

## References

[1] Bhatt S, Gething PW, Brady OJ, Messina JP, Farlow AW, Moyes CL, et al. The global distribution and burden of dengue. Nature. 2013;496(7446):504–7. doi: 10.1038/nature12060 23563266 PMC3651993

[2] Gibbons R V, Vaughn DW. Clinical review Dengue: an escalating problem. Bmj. 2002;324(7353):1563–6. 12089096 10.1136/bmj.324.7353.1563 PMC1123504

[3] World Health Organization (WHO). Dengue and severe dengue. 2021 [cited 2021 Sep 24]. Available from: https://www.who.int/news-room/fact-sheets/detail/dengue-and-severe-dengue.

[4] Lambrechts L, Scott TW, Gubler DJ. Consequences of the expanding global distribution of aedes albopictus for dengue virus transmission. PLoS Negl Trop Dis. 2010;4(5):e646. doi: 10.1371/journal.pntd.0000646 20520794 PMC2876112

[5] Naing C, Ren WY, Man CY, Fern KP, Qiqi C, Ning CN, et al. Awareness of dengue and practice of dengue control among the semi-urban community: A cross sectional survey. J Community Health. 2011;36(6):1044–9. doi: 10.1007/s10900-011-9407-1 21528416

[6] Sharmin S, Glass K, Viennet E, Harley D. Geostatistical mapping of the seasonal spread of under-reported dengue cases in Bangladesh. PLoS Negl Trop Dis. 2018;12(11):1–13. doi: 10.1371/journal.pntd.0006947 30439942 PMC6264868

[7] Sharmin S, Viennet E, Glass K, Harley D. The emergence of dengue in Bangladesh: Epidemiology, challenges and future disease risk. Trans R Soc Trop Med Hyg. 2015;109(10):619–27. doi: 10.1093/trstmh/trv067 26333430

[8] Yunus E Bin, Bangali AM, Mahmood MAH, Rahman MM, Chowdhury AR, Talukder KR. Dengue outbreak 2000 in Bangladesh: From speculation to reality and exercises. Dengue Bull. 2001;25:15–20.

[9] Hossain MA, Khatun M, Arjumand F, Nisaluk A, Breiman RF. Serologic Evidence of Dengue Infection before Onset of. Emerg Infect Dis. 2003;9(11):9–12. 10.3201/eid0911.030117 PMC3035545 14718084

[10] Mone FH, Hossain S, Hasan MT, Tajkia G, Ahmed F. Sustainable actions needed to mitigate dengue outbreak in Bangladesh. Lancet Infect Dis. 2019;19(11):1166–7. doi: 10.1016/S1473-3099(19)30541-9 31657774

[11] Tabassum T, Taylor-Robinson AW. Dengue Serotypes in Bangladesh: Whole Genome Sequencing and Comparative Genomics Facilitates Pathogenesis and Epidemiology Studies and Informs Improved Disease Control. Microbiol Infect Dis. 2019;3(1).

[12] Bashar K, Mahmud S, Asaduzzaman, Tusty EA, Zaman AB. Knowledge and beliefs of the city dwellers regarding dengue transmission and their relationship with prevention practices in Dhaka city, Bangladesh. Public Heal Pract. 2020;1(December):100051. Available from: doi: 10.1016/j.puhip.2020.100051 36101699 PMC9461599

[13] Hasan MM, Sahito AM, Muzzamil M, Mohanan P, Islam Z, Billah MM, et al. Devastating dengue outbreak amidst COVID-19 pandemic in Bangladesh: an alarming situation. Trop Med Health. 2022;50(1):1–5. Available from: 10.1186/s41182-022-00401-y. 35078540 PMC8786620

[14] Directorate General of Health Services Bangladesh. Daily Dengue Status Report. 2022 [cited 2023 May 30]. Available from: https://old.dghs.gov.bd/images/docs/vpr/20221231_dengue_all.pdf.

[15] Directorate General of Health Services Bangladesh. Daily Dengue Status Report. 2023 [cited 2023 May 30]. Available from: https://old.dghs.gov.bd/images/docs/vpr/20230530_dengue_all.pdf.

[16] World Health Organization (WHO). Gglobal strategy for dengue prevention and control 2012–2020. 2012.

[17] Abir T, Ekwudu O, Kalimullah NA, Nur-A Yazdani DM, Mamun A Al, Basak P, et al. Dengue in Dhaka, Bangladesh: Hospital-based cross-sectional KAP assessment at Dhaka North and Dhaka South City Corporation area. PLoS One. 2021;16(3 March):1–17. Available from: doi: 10.1371/journal.pone.0249135 33784366 PMC8009423

[18] Siddiqua M, Alam AN, Muraduzzaman AKM, Tahmina S. NS-1 antigen positive dengue infection and molecular characterization of dengue viruses in a private medical college hospitalin Dhaka, Bangladesh. Bangladesh J Med Sci. 2018;17(4):669–73.

[19] Jeelani S, Sabesan S, Subramanian S. Community knowledge, awareness and preventive practices regarding dengue fever in Puducherry—South India. Public Health. 2015;129(6):790–6. Available from: doi: 10.1016/j.puhe.2015.02.026 25863688

[20] Oki M, Sunahara T, Hashizume M, Yamamoto T. Optimal timing of insecticide fogging to minimize dengue cases: Modeling dengue transmission among various Seasonalities and transmission intensities. PLoS Negl Trop Dis. 2011;5(10). doi: 10.1371/journal.pntd.0001367 22039560 PMC3201920

[21] Dieng H, Saifur RGM, Hassan AA, Che Salmah MR, Boots M, Satho T, et al. Indoor-breeding of Aedes albopictus in northern peninsular Malaysia and its potential epidemiological implications. PLoS One. 2010;5(7). doi: 10.1371/journal.pone.0011790 20668543 PMC2910701

[22] Gubler DJ. Aedes aegypti and Aedes aegypti-Borne Disease Control in the 1990s: Top Down or Bottom Up. Am J Trop Med Hyg. 1989;40(6):571–8. 2472746 10.4269/ajtmh.1989.40.571

[23] Van Benthem BHB, Khantikul N, Panart K, Kessels PJ, Somboon P, Oskam L. Knowledge and use of prevention measures related to dengue in northern Thailand. Trop Med Int Heal. 2002;7(11):993–1000. doi: 10.1046/j.1365-3156.2002.00950.x 12390606

[24] Fang R, Lo E, Lim TW. The 1982 dengue epidemic in Malaysia: epidemiological, serological and virological aspects. Southeast Asian J Trop Med Public Heal. 1984;15(1):51–8. 6740379

[25] Wong LP, AbuBakar S, Chinna K. Community Knowledge, Health Beliefs, Practices and Experiences Related to Dengue Fever and Its Association with IgG Seropositivity. PLoS Negl Trop Dis. 2014;8(5):e2789. doi: 10.1371/journal.pntd.0002789 24853259 PMC4031145

[26] Artwanichakul K, Thiengkamol N, Thiengkamol T. Structural Model of Dengue Fever Prevention and Control Behavior. Eur J Soc Sci. 2012;32(4):485–97.

[27] Haider N, Chang YM, Rahman M, Zumla A, Kock RA. Dengue outbreaks in Bangladesh: Historic epidemic patterns suggest earlier mosquito control intervention in the transmission season could reduce the monthly growth factor and extent of epidemics. Curr Res Parasitol Vector-Borne Dis. 2021;1(July):100063. Available from: doi: 10.1016/j.crpvbd.2021.100063 35284868 PMC8906128

[28] World Health Organization. Dengue—Bangladesh. 2022 [cited 2023 May 15]. Available from: https://www.who.int/emergencies/disease-outbreak-news/item/2022-DON424.

[29] Al-Amin HM, Johora FT, Irish SR, Hossainey MRH, Vizcaino L, Paul KK, et al. Insecticide resistance status of Aedes aegypti in Bangladesh. Parasites and Vectors. 2020;13(1):1–15. Available from: 10.1186/s13071-020-04503-6. 33317603 PMC7734861

[30] Dhar-Chowdhury P, Emdad Haque C, Michelle Driedger S, Hossain S. Community perspectives on dengue transmission in the City of Dhaka, Bangladesh. Int Health. 2014;6(4):306–16. doi: 10.1093/inthealth/ihu032 24981443

[31] Glanz Karen, Barbara K. Rimer KV. Health Behavior and Health Education: Theory, Research, and Practice. 4 th. San Francisco (CA): John Wiley & Sons; 2008. 28–592 p.

[32] Lennon JL. The use of the health belief model in dengue health education. Dengue Bull. 2005;29:217–9.

[33] Chandren JR, Wong LP, AbuBakar S. Practices of dengue fever prevention and the associated factors among the Orang Asli in Peninsular Malaysia. PLoS Negl Trop Dis. 2015;9(8):1–17. 10.1371/journal.pntd.0003954 PMC4534093 26267905

[34] Hossain MI, Alam NE, Akter S, Suriea U, Aktar S, Shifat SK, et al. Knowledge, awareness and preventive practices of dengue outbreak in Bangladesh: A countrywide study. PLoS One. 2021;16(6 June):1–17. Available from: doi: 10.1371/journal.pone.0252852 34111157 PMC8192001

[35] Lim SP. Dengue drug discovery: Progress, challenges and outlook. Antiviral Res. 2019;163(September 2018):156–78. Available from: doi: 10.1016/j.antiviral.2018.12.016 30597183

[36] Prompetchara E, Ketloy C, Thomas SJ, Ruxrungtham K. Dengue vaccine: Global development update. Asian Pacific J Allergy Immunol. 2020;38(3):178–85. doi: 10.12932/AP-100518-0309 30660171

[37] Sardar MIA, Iqubal MKF, Haque ME, Eshita I, Uddin SMS. Change in agriculture due to urbanization at Savar upazila. J Agrofor Environ. 2018;2(1):83–5.

[38] Bangladesh Municipal Development Fund (BMDF). Environmental Assessment Report: Improvement of Road and Drain at Different Location of Savar Pourashava. 2013.

[39] Chareonviriyaphap T, Akratanakul P, Nettanomsak S, Huntamai S. Larval habitats and distribution patterns of Aedes aegypti (Linnaeus) and Aedes albopictus (Skuse), in Thailand. Southeast Asian J Trop Med Public Health. 2003;34(3):529–35. 15115122

[40] Khan MM. Functioning of Local Government (Union Parishad): Legal and Practical Constraints. 2008.

[41] Othman H, Zaini Z-’Izzat I, Karim N, Abd Rashid NA, Abas MBH, Sahani M, et al. Applying health belief model for the assessment of community knowledge, attitude and prevention practices following a dengue epidemic in a township in Selangor, Malaysia. Int J Community Med Public Heal. 2019;6(3):958.

[42] Saied KG, Al-Taiar A, Altaire A, Alqadsi A, Alariqi EF, Hassaan M. Knowledge, attitude and preventive practices regarding dengue fever in rural areas of Yemen. Int Health. 2015;7(6):420–5. doi: 10.1093/inthealth/ihv021 25858280

[43] Banik R, Islam S, Ur M, Pranta R, Rahman QM, Rahman M. Understanding the determinants of COVID- 19 vaccination intention and willingness to pay: findings from a population-based survey in Bangladesh. BMC Infect Dis. 2021;21(892):1–15. doi: 10.1186/s12879-021-06406-y 34465297 PMC8406014

[44] Mutsuddy P, Tahmina Jhora S, Shamsuzzaman AKM, Kaisar SMG, Khan MNA, Dhiman S. Dengue Situation in Bangladesh: An Epidemiological Shift in terms of Morbidity and Mortality. Can J Infect Dis Med Microbiol. 2019;2019(October):2017–22. doi: 10.1155/2019/3516284 30962860 PMC6431455

[45] Udayanga L, Gunathilaka N, Iqbal MCM, Pahalagedara K, Amarasinghe US, Abeyewickreme W. Socio-economic, Knowledge Attitude Practices (KAP), household related and demographic based appearance of non-dengue infected individuals in high dengue risk areas of Kandy District, Sri Lanka. BMC Infect Dis. 2018;18(1):1–14. 29466952 10.1186/s12879-018-2995-y PMC5822474

[46] Taksande A, Lakhkar B. Knowledge, attitude and practice (KAP) of Dengue Fever in the Rural area of Central India. Shiraz E Med J. 2013;13(4):146–57.

[47] Selvarajoo S, Liew JWK, Tan W, Lim XY, Refai WF, Zaki RA, et al. Knowledge, attitude and practice on dengue prevention and dengue seroprevalence in a dengue hotspot in Malaysia: A cross-sectional study. Sci Rep. 2020;10(1):1–13. Available from: 10.1038/s41598-020-66212-5. 32533017 PMC7293214

[48] Harapan H, Rajamoorthy Y, Anwar S, Bustamam A, Radiansyah A, Angraini P, et al. Knowledge, attitude, and practice regarding dengue virus infection among inhabitants of Aceh, Indonesia: A cross-sectional study. BMC Infect Dis. 2018;18(1):1–16. 29486714 10.1186/s12879-018-3006-z PMC5830327

[49] Alyousefi TAA, Abdul-Ghani R, Mahdy MAK, Al-Eryani SMA, Al-Mekhlafi AM, Raja YA, et al. A household-based survey of knowledge, attitudes and practices towards dengue fever among local urban communities in Taiz Governorate, Yemen. BMC Infect Dis. 2016;16(1):1–9. Available from: 10.1186/s12879-016-1895-2. 27717333 PMC5054547

[50] Dégallier N, Vilarinhos PT, Carvalho MS de, Knox MB, Jr JC. People’s knowledge and practice about dengue, its vectors, and control means in Brasilia (DF), Brazil: its relevance with entomological factors. J Am Mosq Control Assoc. 2000;16(2):114–23. 10901634

[51] Islam S, Haque CE, Hossain S, Walker D. Association among ecological and behavioural attributes, dengue vector and disease control: U cross-sectional study of the city of Dhaka, Bangladesh. Int Health. 2020;12(5):444–54. 31782495 10.1093/inthealth/ihz079 PMC7443721

[52] Dhaka Tribune. Bangladesh reports 70 more dengue cases in 24 hours. 2021 [cited 2021 Nov 5]. Available from: https://www.dhakatribune.com/bangladesh/2021/07/12/bangladesh-reports-70-more-dengue-cases-in-24-hours.

[53] New Age. 1 more dies of dengue, 157 others hospitalised. 2021 [cited 2021 Nov 5]. Available from: https://www.newagebd.net/article/153685/1-more-dies-of-dengue-157-others-hospitalised.

[54] Shuaib F, Todd D, Campbell-Stennett D, Ehiri J, Jolly PE. Knowledge, attitudes and practices regarding dengue infection in Westmoreland, Jamaica. West Indian Med J. 2010;59(2):139–146. 21132094 PMC2996104

[55] Cernters for Disease Control and Prevention (CDC). Symptoms of COVID-19. 2021 [cited 2021 Nov 19]. Available from: https://www.cdc.gov/coronavirus/2019-ncov/symptoms-testing/symptoms.html.

[56] Dhimal M, Aryal KK, Dhimal ML, Gautam I, Singh SP, Bhusal CL, et al. Knowledge, attitude and practice regarding dengue fever among the healthy population of highland and lowland communities in Central Nepal. PLoS One. 2014;9(7):e102028. doi: 10.1371/journal.pone.0102028 25007284 PMC4090170

[57] Teo D, Ng LC, Lam S. Is dengue a threat to the blood supply? Transfus Med. 2009;19(2):66–77. doi: 10.1111/j.1365-3148.2009.00916.x 19392949 PMC2713854

[58] Wilder-Smith A, Chen LH, Massad E, Wilson ME. Threat of dengue to blood safety in dengue-endemic countries. Emerg Infect Dis. 2009;15(1):8–11. doi: 10.3201/eid1501.071097 19116042 PMC2660677

[59] Wong LP, Shakir SMM, Atefi N, AbuBakar S. Factors affecting dengue prevention practices: Nationwide survey of the Malaysian public. PLoS One. 2015;10(4):1–16. doi: 10.1371/journal.pone.0122890 25836366 PMC4383514

[60] Itrat A, Khan A, Javaid S, Kamal M, Khan H, Javed S, et al. Knowledge, awareness and practices regarding dengue fever among the adult population of dengue hit cosmopolitan. PLoS One. 2008;3(7):1–6. doi: 10.1371/journal.pone.0002620 18612437 PMC2440812

[61] Castro M, Sańchez L, Peŕez D, Sebrango C, Shkedy Z, Van Der Stuyft P. The relationship between economic status, knowledge on dengue, risk perceptions and practices. PLoS One. 2013;8(12):6–11. doi: 10.1371/journal.pone.0081875 24349145 PMC3861357

[62] Koenraadt CJM, Tuiten W, Sithiprasasna R, Kijchalao U, Jones JW, Scott TW. Dengue knowledge and practices and their impact on Aedes aegypti populations in Kamphaeng Phet, Thailand. Am J Trop Med Hyg. 2006;74(4):692–700. 16607007

[63] Cerrato J, Cifre E. Gender inequality in household chores and work-family conflict. Front Psychol. 2018;9(August):1–11. doi: 10.3389/fpsyg.2018.01330 30123153 PMC6086200

[64] Diaz-Quijano FA, Martínez-Vega RA, Rodriguez-Morales AJ, Rojas-Calero RA, Luna-González ML, Díaz-Quijano RG. Association between the level of education and knowledge, attitudes and practices regarding dengue in the Caribbean region of Colombia. BMC Public Health. 2018;18(1):1–10. doi: 10.1186/s12889-018-5055-z 29338712 PMC5771071

[65] Hairi F, Ong CHS, Suhaimi A, Tsung TW, Bin Anis Ahmad MA, Sundaraj C, et al. A Knowledge, Attitude and Practices (KAP) Study on Dengue among Selected Rural Communities in the Kuala Kangsar District. Asia-Pacific J Public Heal. 2003;15(1):37–43. 10.1177/101053950301500107 14620496

