# Supplementary material for: Public knowledge, belief, and preventive practices regarding dengue: Findings from a community-based survey in rural Bangladesh
Source: PLoS Negl Trop Dis. 2023 Dec 7;17(12):e0011778. doi: 10.1371/journal.pntd.0011778 (PMC10754436; doi:10.1371/journal.pntd.0011778)
Supplement: S1 Table — (DOCX) [file pntd.0011778.s001.docx]

**S1 Table.** Distribution of the Health Belief Model (HBM) constructs.

| **Statement** | **Percentage (%)** | | | | | |
| --- | --- | --- | --- | --- | --- | --- |
|  | **Strongly agree** | | **Agree** | **Disagree** | **Strongly disagree** | |
| ***Perceived Severity*** | | | | | | |
| I feel that dengue is very dangerous. | 1.75 | | 10.72 | 7.98 | 58.1 | |
| I do not think dengue fever can caused death. | 12.97 | | 16.46 | 7.98 | 51.37 | |
| I'm afraid of dengue, even though there are various medical facilities to treat me. | 6.23 | | 20.95 | 11.72 | 53.62 | |
| ***Perceived Susceptibility*** | | | | | | |
| I am likely to be infected with Dengue if I get bitten by mosquitoes frequently. | | 3.99 | 15.21 | 10.22 | | 61.1 |
| I think, my chance of getting infected with dengue in the next few months is high. | | 3.74 | 18.45 | 36.41 | | 37.41 |
| I am unlikely to get dengue infection because I already had it before | | 20.45 | 46.13 | 17.96 | | 14.71 |
| ***Perceived Barriers*** | | | | | | |
| I don’t have sufficient knowledge on dengue and its prevention | 7.98 | | 33.67 | 17.96 | | 35.41 |
| My residential area has sufficient areas where mosquitoes can breed. | 2.74 | | 14.96 | 14.71 | | 62.34 |
| I have access to medical facilities in case of dengue. | 3.24 | | 12.97 | 10.72 | | 64.09 |
| I think Government agencies are responsible for eradication of mosquitoes. | 2.00 | | 17.96 | 15.21 | | 52.62 |
| ***Self-efficacy*** | | | | | | |
| I can assure that there are no breeding spots in the neighborhood of my house. | 1.25 | | 2.74 | 8.98 | | 72.32 |
| I always keep clean my surround area. | 0.50 | | 0.75 | 5.24 | | 76.56 |
| I can engage with the community to increase participation and mobilization in the fight against vectors. | 1.00 | | 0.75 | 7.98 | | 73.82 |
| ***Cues to action*** | | | | | | |
| I think community people are not aware in taking preventive measures against dengue | 1.50 | | 17.96 | 12.97 | | 55.86 |
| I feel that the government measures in controlling dengue is not effective. | 1.25 | | 9.98 | 18.20 | | 54.86 |
