# Supplementary material for: Public knowledge, belief, and preventive practices regarding dengue: Findings from a community-based survey in rural Bangladesh
Source: PLoS Negl Trop Dis. 2023 Dec 7;17(12):e0011778. doi: 10.1371/journal.pntd.0011778 (PMC10754436; doi:10.1371/journal.pntd.0011778)
Supplement: S1 File — (DOCX) [file pntd.0011778.s002.docx]

**Research title:** Public knowledge, belief, and preventive practices regarding dengue: Findings from a community-based survey in rural Bangladesh

**Full questionnaire**

| ***Section: A***  ***Socio-demographics, housing, surrounding environment,***  ***and dengue-related experience*** | | | | | | |
| --- | --- | --- | --- | --- | --- | --- |
| A.1 | What is your age? | _________________years | | |  | |
| A.2 | Gender | [1] = Male | | | [2] = Female | |
| A.3 | Marital Status | [1] = Unmarried | [2] = Married | | | [2] = Others |
| A.4 | Please mention your highest education attainment | [1] = No formal education  [3] = Secondary level  [5] = Graduation  [7] = Others | | | [2] = Primary level  [4] = Higher secondary  [6] = Post graduation | |
|  |  | **[If you select others, please specify below:**  **______________________________________]** | | | | |
| A.5 | What is your occupation? | [1] = Housewife  [3] = Govt. employee  [5] = Daily wager  [7] = Unemployed | | | [2] = Student  [4] = Private employee  [6] = Self-employed  [8] = Others | |
|  |  | **[If you select others, please specify below:**  **______________________________________]** | | | | |
| A.6 | Type of family | [1] = Nuclear [2] = Joint [3] = Extended | | | | |
| A.7 | What is your monthly family income? | [1] = Less than 20000 BDT  [3] = 30000-40000 BDT | | | [2] = 20000-30000 BDT  [4] = More than 40000 BDT | |
| A.8 | Types of house | [1] = Building  [3] = Tin-shade | | | [2] = Half-building  [4] = Others | |
|  |  | **[If you select others, please specify below:**  **_______________________________________]** | | | | |
| A.9 | Mention the amount/density of plants or vegetation in your house. | [1] = None  [3] = Moderate | | [2] = Low  [4] = A lot | | |
| A.10 | Presence of any types of water reservoir in surrounding your house? | [1] = Yes | | [2] = No | | |
| A.11 | Mention the quantity/amount of mosquitoes in the neighborhood. | [1] = Not at all  [3] = Moderate | | [2] = Low  [4] = Severe | | |
| A. 12 | Have you ever had dengue fever? | [1] = Yes | | [2] = No  **[**If you select **No**, then go to **A. 14]** | | |
| A. 13 | Have you been hospitalized because of dengue? | [1] = Yes | | [2] = No | | |
| **A. 14** | Has anyone living in your household (family member) experienced dengue fever? | [1] = Yes | | [2] = No | | |
| A. 15 | Do you often travel or been in an area where dengue has been reported? | [1] = Yes [2] = No [3] = Don’t Know | | | | |

| ***Section: B***  ***Knowledge about Dengue*** | | | | | | | |
| --- | --- | --- | --- | --- | --- | --- | --- |
| **Statements** | | | | **Yes** | **No** | **Don’t Know** |  |
| **Knowledge about Dengue and Aedes mosquito** | | | |  |  |  |  |
| B.1 | Dengue is caused by a virus. | | | [1] | [2] | [3] |  |
| B.2 | Dengue is transmitted by mosquito bite. | | | [1] | [2] | [3] |  |
| B.3 | Aedes aegypti and Aedes albopictus are main vectors for dengue in Bangladesh. | | | [1] | [2] | [3] |  |
| B.4 | Aedes mosquitos have black and white stripes on its leg and body | | | [1] | [2] | [3] |  |
| B.5 | Aedes mosquito breeds in clean and stagnant water | | | [1] | [2] | [3] |  |
| B.6 | Aedes mosquito prefers to live in the house or building rather than in natural wetlands | | | [1] | [2] | [3] |  |
| B.7 | Aedes mosquito prefers to live in places with a lot of plants | | | [1] | [2] | [3] |  |
| B.8 | Aedes mosquitoes mainly bite during daytime (dusk and dawn). | | | [1] | [2] | [3] |  |
| **Knowledge about the transmission of Dengue** | | | | | | | |
| B.9 | The Aedes mosquitoes biting an infected person can spread it to another person | | | [1] | [2] | [3] |  |
| C.10 | How Dengue is transmitted? | | Human to human contact | [1] | [2] | [3] |  |
|  |  |  | Through air | [1] | [2] | [3] |  |
|  |  |  | Blood transfusion | [1] | [2] | [3] |  |
|  |  |  | Needle stick injury | [1] | [2] | [3] |  |
|  |  |  | Sharing of food/ clothes with the patient | [1] | [2] | [3] |  |
|  |  |  | Human to human contact | [1] | [2] | [3] |  |
| C.11 | Aedes mosquitoes’ eggs can contain the dengue virus | | | [1] | [2] | [3] |  |
| C.12 | A person who has had dengue fever cannot get the infection again | | | [1] | [2] | [3] |  |
| C.13 | Dengue epidemic occurs only during the rainy season | | | [1] | [2] | [3] |  |
| **Knowledge about the signs and symptoms of Dengue** | | | | | | | |
| B.14 | What are the common sign and symptoms of dengue? | High grade fever | | [1] | [2] | [3] |  |
|  |  | Chills | | [1] | [2] | [3] |  |
|  |  | Skin rash | | [1] | [2] | [3] |  |
|  |  | Muscle pain | | [1] | [2] | [3] |  |
|  |  | Bone/Joint pain | | [1] | [2] | [3] |  |
|  |  | Nausea and vomiting | | [1] | [2] | [3] |  |
|  |  | Headache | | [1] | [2] | [3] |  |
|  |  | Small bleeding at gum and nose | | [1] | [2] | [3] |  |
|  |  | Diarrhea | | [1] | [2] | [3] |  |
|  |  | Pain behind eyes | | [1] | [2] | [3] |  |
| **Knowledge about preventive measures** | | | | | | | |
| B.15 | What measures you should take to prevent Dengue? | Eliminate mosquito breeding spots weekly | | [1] | [2] | [3] |  |
|  |  | Weekly change of stagnant water (pet bowls, vases) | | [1] | [2] | [3] |  |
|  |  | Put Abate/chemical in water containers | | [1] | [2] | [3] |  |
|  |  | Covering water containers that can retain water | | [1] | [2] | [3] |  |
|  |  | Emptying or drying out containers around the house | | [1] | [2] | [3] |  |
|  |  | Proper disposal of items that can retain water | | [1] | [2] | [3] |  |
| **Knowledge about treatment, curability, and precautionary measures for people infected with dengue** | | | | | | | |
| B.16 | What measures can be for people infected with dengue? | No specific treatment | | [1] | [2] | [3] |  |
|  |  | Medication (e.g., Paracetamol) | | [1] | [2] | [3] |  |
|  |  | Taking rest | | [1] | [2] | [3] |  |
|  |  | Drinking plenty of fluids | | [1] | [2] | [3] |  |
|  |  | Vaccination | | [1] | [2] | [3] |  |

|  | ***Section: C***  ***Health Belief Model*** | | | | | | | | | | |
| --- | --- | --- | --- | --- | --- | --- | --- | --- | --- | --- | --- |
|  | Statement | Strongly agree | | | Agree | | | Disagree | | | Strongly disagree |
| **Perceived Severity** | | | | | | | | | | | |
| C.1.1 | I feel that dengue is very dangerous. | [1] | | | [2] | | | [3] | | | [4] |
| C.1.2 | I do not think dengue fever can caused death. | [1] | | | [2] | | | [3] | | | [4] |
| C.1.3 | I'm afraid of dengue, even though there are various medical facilities to treat me. | [1] | | | [2] | | | [3] | | | [4] |
| **Perceived Susceptibility** | | | | | | | | | | | |
| C.2.1 | I am likely to be infected with Dengue if I get bitten by mosquitoes frequently. | | [1] | | | [2] | | | [3] | | [4] |
| C.2.2 | I think, my chance of getting infected with dengue in the next few months is high. | | [1] | | | [2] | | | [3] | | [4] |
| C.2.3 | I am unlikely to get dengue infection because I already had it before | | [1] | | | [2] | | | [3] | | [4] |
| **Perceived Barriers** | | | | | | | | | | | |
| C.3.1 | I don’t have sufficient knowledge on dengue and its prevention | | | [1] | | | [2] | | | [3] | [4] |
| C.3.2 | My residential area has sufficient areas where mosquitoes can breed. | | | [1] | | | [2] | | | [3] | [4] |
| C.3.3 | I have access to medical facilities in case of dengue. | | | [1] | | | [2] | | | [3] | [4] |
| C.3.4 | I think Government agencies are responsible for eradication of mosquitoes. | | | [1] | | | [2] | | | [3] | [4] |
| **Self-efficacy** | | | | | | | | | | | |
| C.4.1 | I can assure that there are no breeding spots in the neighborhood of my house. | | | [1] | | | [2] | | | [3] | [4] |
| C.4.2 | I always keep clean my surround area. | | | [1] | | | [2] | | | [3] | [4] |
| C.4.3 | I can engage with the community to increase participation and mobilization in the fight against vectors. | | | [1] | | | [2] | | | [3] | [4] |
| **Cues to action** | | | | | | | | | | | |
| C.5.1 | I think community people are not aware in taking preventive measures against dengue | | | [1] | | | [2] | | | [3] | [4] |
| C.5.2 | I feel that the government measures in controlling dengue is not effective. | | | [1] | | | [2] | | | [3] | [4] |

| ***Section: D***  ***Practices Towards Dengue Prevention*** | | | | | | | |
| --- | --- | --- | --- | --- | --- | --- | --- |
| Statements | | | Not at all | Rarely | Sometimes | Often | Not applicable |
| **Prevention of mosquito breeding** | | | | | | | |
| D.1 | **Cover** all water containers used for storing water in or outside the house | | [1] | [2] | [3] | [4] | [5] |
| D.2 | Change **stored water** i.e water in water tank, pails, bath tub, flower vases, money plant, **plates that support plant pots, refrigerator drip tray, plastic container on the bottom of kitchen cabinet/table legs (for controlling ants), etc.** | | [1] | [2] | [3] | [4] | [5] |
| D.3 | **Put Abate or chemical** in water storage containers | | [1] | [2] | [3] | [4] | [5] |
| D.4 | **Examine for mosquito larvae** in containers for storing water, eg. pails, flower vases, water in money plant, plates that support plant pots, refrigerator drip tray, plastic container on the bottom of kitchen cabinet/table legs, etc. | | [1] | [2] | [3] | [4] | [5] |
| D.5 | **Clear out debris** that may **block water flow** in drain or **roof** gutters | | [1] | [2] | [3] | [4] | [5] |
| D.6 | **Proper disposal of items** **that can collect rain water** (i.e. bucket, discarded item such as cans, cups,bottles, food container, linolium, old tyres etc.) | | [1] | [2] | [3] | [4] | [5] |
| D.7 | Proper **disposal of household garbage** | | [1] | [2] | [3] | [4] | [5] |
| D.8 | **Clean up surrounding house area** | | [1] | [2] | [3] | [4] | [5] |
| D.9 | Take mosquito preventive measures before going on **long holidays** such as covering all water containers, emptying water containers etc. | | [1] | [2] | [3] | [4] | [5] |
| **Prevention of mosquito bites** | | | | | | | |
| D.10 | | Sleep in mosquito net or have mosquito screens on windows | [1] | [2] | [3] | [4] | [5] |
| D.11 | | Use mosquito coil, electric mosquito mat,liquid vaporizer, mosquito bulb, or mosquito trap | [1] | [2] | [3] | [4] | [5] |
| D.12 | | dark places with an insecticidal spray | [1] | [2] | [3] | [4] | [5] |
| D.13 | | Use mosquito repellent on body | [1] | [2] | [3] | [4] | [5] |
| D.14 | | Avoid dark areas in the home where there is no light and no wind | [1] | [2] | [3] | [4] | [5] |
| D.15 | | Wear long-sleeved shirts and pants to avoid mosquito bites | [1] | [2] | [3] | [4] | [5] |
| D.16 | | Wear bright color clothes to avoid mosquito bites | [1] | [2] | [3] | [4] | [5] |
| **Prevention of dengue transmission** | | | | | | | |
| D.17 | | Take measures to prevent mosquitoes from biting a dengue patient | [1] | [2] | [3] | [4] | [5] |

**Thank you**
